# Janus Kinase Inhibitors and Cell Therapy

**DOI:** 10.3389/fimmu.2021.740847

**Published:** 2021-08-31

**Authors:** Amer Assal, Markus Y. Mapara

**Affiliations:** ^1^Department of Medicine, Bone Marrow Transplantation and Cell Therapy Program, Columbia University Irving Medical Center, New York, NY, United States; ^2^Columbia Center for Translational Immunology, Columbia University, New York, NY, United States

**Keywords:** JAK - STAT signaling pathway, graft-versus-host disease (GvHD), cytokine release syndrome (CRS), transplantation, cell therapy (CT)

## Abstract

Cellular therapies such as allogeneic hematopoietic stem cell transplantation (HSCT) and immune-effector cell therapy (IECT) continue to have a critical role in the treatment of patients with high risk malignancies and hematologic conditions. These therapies are also associated with inflammatory conditions such as graft-*versus*-host disease (GVHD) and cytokine release syndrome (CRS) which contribute significantly to the morbidity and mortality associated with these therapies. Recent advances in our understanding of the immunological mechanisms that underly GVHD and CRS highlight an important role for Janus kinases (JAK). JAK pathways are important for the signaling of several cytokines and are involved in the activation and proliferation of several immune cell subsets. In this review, we provide an overview of the preclinical and clinical evidence supporting the use of JAK inhibitors for acute and chronic GVHD and CRS.

## Introduction

Allogeneic hematopoietic stem cell transplantation (HSCT) continues to grow as a field owing to its curative potential for a variety of hematologic conditions and malignancies (1). Recent advances in immune effector cell therapy (IECT) using chimeric antigen-receptor T (CART) cells have introduced new possibilities and challenges in the treatment of patients with hematologic malignancies (1). Graft-*versus*-host disease (GVHD), both acute and chronic, is a common complication of HSCT and contributor to morbidity and mortality thus limiting its therapeutic potential (2). GVHD incidence, both acute and chronic, is >50% and 7-9% of deaths post-transplant are attributed to GVHD in matched sibling HSCT and 9-10% in unrelated donor HSCT (2–4). Acute GVHD (aGVHD) occurs when donor-derived T cells in the donated graft recognize host antigens as foreign (5). The target antigens of donor-derived T cells include human leukocyte antigen (HLA) molecules, both class I and class II (5). HLA proteins are highly polymorphic and encoded by the major histocompatibility complex (MHC) genes. Donor T cells may also recognize host minor histocompatibility antigens (mHA) contributing to aGVHD. Chronic GVHD (cGVHD) pathophysiology is more complex to model and study; a proposed model suggests that cGVHD is caused by early inflammation due to tissue injury, followed by chronic inflammation, thymic injury and dysregulated B and T cells all leading to tissue repair with fibrosis (6). Several factors have been shown to increase the risk of GVHD and these include donor/recipient HLA mismatch, increased age, sex, conditioning regimen intensity, and donor graft source whether mobilized peripheral blood stem cells or bone marrow (7, 8).

Immune suppression with corticosteroids, with or without a calcineurin inhibitor or sirolimus, remains the mainstay of treatment for both acute and chronic GVHD, which has changed very little over the past 40 years (9–11). One of the limitations affecting reproducibility and generalizability of GVHD clinical trial results has been a lack of consistency in diagnosing and grading GVHD (12). The efficacy of corticosteroids in the treatment of aGVHD is limited with response rates ranging from 30 to 64% (9, 13). Treatment related mortality remains high even in responders and is markedly increased in steroid refractory aGVHD (SR-aGVHD) (14). cGVHD outcomes are also poor despite treatment as the majority of cases require multiple lines of therapy and only a third of cases achieve long term remissions off of immune suppression (15).

Similarly, IECT is complicated by cytokine release syndrome (CRS), which is an inflammatory condition that can be life-threatening and require intensive care (16, 17). The incidence of CRS varies by the cell product used as well as by the malignancy treated. Patients with acute lymphoblastic leukemia (ALL) have a higher reported rate of CRS than large B cell lymphoma (LBCL) in their respective registration trials with tisagenlecleucel (18, 19). Axicabtagene ciloleucel, which contains a CD28 costimulatory domain rather than 4-1BB used in tisagenlecleucel, also reported higher rates of CRS in the registration trial (20). Risk factors suggested for the development of CRS include higher disease burden, higher cell dose infused, lymphodepleting chemotherapy selection, cell product used, a low pre-treatment platelet count, and the CD4/CD8 T cell ratio (16, 17). Comparison of CRS rates across trials can be challenging owing to different CRS grading systems, however increased adoption of the American Society of Transplant and Cellular Therapy (ASTCT) grading schema may help address this (21). The pathogenesis of CRS is related to the activation of CART cells as well as other immune cells such as those of the monocyte/macrophage lineage (16, 17). Elevation in several cytokines and inflammatory mediators are noted during CRS contributing to endothelial activation, capillary leak, and coagulopathy. Treatment of CRS includes supportive care measures for lower grades, and tocilizumab, an IL-6 receptor antagonist that is the only FDA-approved therapy for CRS, for grades 2 or greater. Corticosteroids are also used for higher grade CRS particularly when it is associated with neurotoxicity (22). Optimizing the toxicity and financial impact of IECT remains a challenge as more centers move towards outpatient administration (23).

Targeting the Janus kinase (JAK) - signal transducer and activator of transcription (STAT) pathway through JAK inhibition has emerged as a promising therapeutic strategy for GVHD and CRS. Insights into the pathogenesis of GVHD demonstrate a necessary role for signaling through the JAK/STAT pathway, particularly STAT1 and STAT3 (24–27). This is supported by clinical efficacy of JAK inhibitors in the treatment of acute and chronic GVHD (28–32). The FDA approval of ruxolitinib, a JAK1/2 inhibitor, represents a major advance in the treatment of SR-aGVHD (31). Furthermore, the JAK/STAT pathways are critical for cytokine signaling suggesting a potential role for JAK inhibition in the management of CRS (33). The JAK1 inhibitor itacitinib is currently being studied for CRS prophylaxis in recipients of IECT (34). In this review, we will present an overview of the role of JAK/STAT pathways in GVHD and inflammatory conditions relevant to cell therapies such as CRS and present recent clinical developments in the field.

## JAK/STAT Pathway in GVHD

The identification of several cytokines as key players in the pathogenesis of GVHD (such as interferon-γ (IFN-γ), tumor necrosis factor (TNF) suggested early on that targeting pathways involved the in signal transduction of these cytokines may be promising targets for therapeutic intervention. Early evidence linking GVHD and cytokine signaling through the JAK/STAT pathways was found by expression profiling studies (35) and results from our groups detecting activation of STAT1/3 activation in GVHD target organs (24, 25) and in donor T cells (26) in murine models of GVHD. In addition, HDAC-inibition -dependent mitigation of GVHD was associated with reduced STAT1 activation (24). Our laboratory was the first to show that disruption of the JAK/STAT1 signaling pathway in donor T cells prevented development of GVHD in minor Ag-mismatched GVHD and mitigated GVHD in fully-MHC mismatched GVHD (MA) (26). Furthermore, we could show that the observed effect was achieved by blocking IFNγ-R signaling rather than IFNα-R signaling (25).

Reduced alloantigen induced activation and proliferation was seen in STAT1-deficient donor T cells, and correlated with CD4+CD25+Foxp3+ Treg expansion (26). Our findings were confirmed and further expanded upon by the labs of Choi and DiPersio showing similar results using IFN-γ-receptor knock-out donors demonstrating that pharmaceutical targeting of JAK1/2 signaling is highly effective in preventing GVHD while retaining GVL-responses (36, 37). Thus, ruxolitinib treatment was found to ameliorate GVHD in MHC-mismatched murine models (36–38). Decreased T cell expansion as well as a higher frequency of CD4+Foxp3+ Tregs and lower frequencies of central memory T cells were observed in treated mice (38, 39). *In vitro* studies of CD4+ T cells stimulated with allogeneic dendritic cells (DC) showed decreased T cell expansion and cytokine production in presence of ruxolitinib treatment and CD4+ STAT3 phosphorylation (38). Baricitinib, another JAK1/2 inhibitor, was also shown to be effective in blocking GVHD in MHC-mismatched murine models as well as treating ongoing GVHD (40).

Another aspect of JAK/STAT involvement in GVHD pathogenesis involves its role in chemokine-mediated T cell trafficking to target organs. IFN-γ receptor deficient conventional T cells were found to be defective in trafficking to target organs and exhibited reduced CXCR3 expression, a phenotype that was replicated by the use of ruxolitinib or momelotinib as JAK1/JAK2 inhibitors (37). Further work by the same group demonstrated a preservation of the graft-*versus*-leukemia effect in 2 different murine MHC-mismatched allogeneic HSCT models using either a myeloid or lymphoid murine leukemia models (36). Similar results were reported by another group where ruxolitinib-treated mice exhibited decreased T cell and macrophage migration to the skin, small intestine, and liver (41). Decreased expression of CXCR3 on splenic CD4+ and CD8+ T cells was also observed.

The JAK/STAT pathway also has a role in modulating APCs. Ruxolitinib and the JAK1/JAK3 inhibitor tofacitinib suppressed the inflammatory phenotype of macrophages isolated from patients with rheumatoid arthritis (42). IFN-α and IFN-γ mediated STAT1 and STAT3 phosphorylation was blocked by JAK inhibitors in macrophages. Furthermore, TNF-dependent STAT1 activation, STAT1 expression and IFN-dependent genes were blocked by JAK inhibitors. JAK1/2 inhibition with ruxolitinib was also shown to affect DC function by impairing monocyte differentiation, DC activation and DC-dependent T cell activation (43). JAK/STAT inhibition may be particularly effective in patients with a MicroRNA-146a CC polymorphism which leads to lower levels of miR-146a and subsequently increased JAK/STAT pathway signaling and MHC II expression in DC (44). Baricitinib was also shown to exert effects on antigen presenting cells (APC) as decreased expression of MHC II, CD80/86 and PD-L1 was noted on recipient CD11c+ and B220+ APCs (40). Neutrophils, which are the first cells to reach sites of tissue injury after conditioning chemotherapy, migrate to the mesenteric lymph nodes, increase expression of MHC II, and may present antigen to T cells (45). JAK/STAT inhibition with ruxolitinib may attenuate the role of neutrophils in mediating GVHD (45).

While significant evidence supports the role of multi-kinase inhibitors that target more than 1 JAK protein, selective JAK1 or JAK2 inhibition has also been shown to be effective in GVHD models. Itacitinib, a selective JAK1 inhibitor currently being studied in clinical trials, has been shown to inhibit weight loss and improve GVHD scores without impacting engraftment in mismatched MHC mouse models (30). On the other hand, JAK2-/- donor T cells also lead to attenuated GVHD without impacting graft-*versus-*leukemia effect (46). JAK2-/- T cells exhibit decreased Th1 polarization and increased Treg and Th2 polarization. Pacritinib, a JAK2 selective inhibitor, significantly reduced GVHD in murine models, induced a Th2 polarization in human T cells, and spared Tregs.

Considering the role of the JAK/STAT pathways in T cell activation and expansion, APC function, and Tregs expansion, JAK inhibitors are well positioned to also have a role in cGVHD treatment. Tregs frequency is reduced in patients with cGVHD (47), and treatment with low dose IL-2 ameliorated cGVHD in patients with glucocorticoid-refractory cGVHD which was associated with Treg expansion and an increased Treg to conventional T cell ratio (48). Patients with active cGVHD have lower frequencies of circulating T follicular helper cells which are skewed towards a highly activated profile and also have higher levels of CXCL13 (49). Furthermore, in a murine model of sclerodermatous cGVHD, donor macrophages mediated cGVHD-like manifestations (50). Recent experimental evidence support the role of JAK1/2 in cGVHD as inhibition with ruxolitinib was shown to attenuate cGVHD in a murine sclerodermatous murine model where a decrease in the frequency of effector CD4+ T cells and CD11b+ macrophages, and IFN-γ producing CD4+ T cells was noted as well as an expansion in Tregs (51). Ruxolitinib suppressed IFN-γ production by CD4+ T cells and monocyte chemoattractant protein (MCP)-1 from CD11b+ macrophages, the proliferation of these cells, as well as the migration of a macrophage cell line in response to IFN-γ.

## JAK/STAT Pathway and CRS

As the JAK/STAT pathways play an important role in immune function and modulation, targeting these pathways in hyperinflammatory conditions such as CRS is a reasonable consideration. The activation of several immune cell subsets is responsible for the cytokine profile of CRS. Elevations in IFN-γ, IL6, IL8, soluble interleukin 2 receptor (sIL2R)-α, sgp130, soluble IL6 receptor (sIL6R), MCP1, MIP1α, MIP1β, and granulocyte-macrophage colony-stimulating factor (GM-CSF) were noted in ALL recipients of IECT who developed severe CRS (52). Interestingly, a nearly identical pattern of cytokine elevation was noted in patients with hematophagocytic lymphohistiocytosis (HLH). IL-6 production is derived from monocytes in response to CART cell recognition of their targets (53, 54). IL-1 secretion preceded IL-6 secretion in a mouse of IECT and IL-1 blockade is emerging as a promising strategy for CRS and neurotoxicity management (54–56).

Considering the similarity in the pathophysiology of CRS and HLH, lessons learned from experimental and therapeutic studies in HLH may be applicable to CRS. Ruxolitinib administration improved survival and physiological parameters in murine models of HLH, decreased levels of phosphorylated STAT1 in peripheral blood white blood cells, decreased serum levels of IL-6, TNF-α, MCP-1, CXCL10, and soluble IL-2 receptor, and reduced tissue infiltration (57). Ruxolitinib was found to act in INF-γ dependent and independent pathways in another study which showed similar findings including a lowering CD8+ T cell and neutrophil infiltrations of organs, dampening CD8+ T cell activation, and decreased production of TNF-α, IFN-γ by T cells, and lower levels of TNF-a, IL-6, IL-12, CXCL10, IL-1b, GM-CSF, MIP-1a and G-CSF (58). In a study of multiple models of hyperinflammation, ruxolitinib was effective in reducing inflammation including a murine model of HLH as in the preceding study, and reduced IL-6 production by macrophages *in vitro* (59). Ruxolitinib may also sensitize CD8+ T cells to dexamethasone which is commonly used as therapy in hyperinflammatory syndromes (60). Ruxolitinib has since been successfully used to treat patients with HLH (61–63), although cases of relapsed disease in lymphoma-associated HLH were also reported in the setting of ruxolitinib treatment (64). These experimental and clinical findings support the targeting of JAK/STAT pathways in hyperinflammatory syndromes including potentially CRS which overlaps with HLH in its pathophysiology (52). Itacitinib, a JAK1 selection inhibitor, was studied *in vitro* and *in vivo* in IECT models (65). Itacitinib successfully reduced cytokine levels associated with CRS in a murine model of hyperinflammation, reduced IL-6 production by macrophages *in vitro* and *in vivo*, reduced cytokines production by CART cells, and had no impact on CD8+ T cell or CART cell expansion or target lysis at lower doses that are pharmacologically relevant. A recent report studied ruxolitinib as CRS prophylaxis in patients with relapse-refractory acute myeloid leukemia who were being treated with a CD123 x CD3 bispecific molecule (66). Cytokine analysis showed a significant reduction in levels of IL-4, IL-12p70, IL-13, IL-15, IL-17A, IFN-α2, but higher levels of GM-CSF. However, the incidence and severity of CRS events were similar. Only a small number of patients were treated with ruxolitinb (10 patients). Itacitinib is currently being studied as CRS prophylaxis in an ongoing phase II study (34).

## Clinical Efficacy of JAK Inhibitors in the Treatment of GVHD

### Studies in Refractory GVHD

In light of supporting preclinical evidence and the lack of effective alternatives, JAK inhibitors were used as salvage therapies in GVHD with great success (28, 38). Of the earliest reports of JAK inhibitors for the treatment of GVHD were published by Zeiser et al. who described outcomes after ruxolitinib therapy for GVHD in patients from multiple stem cell transplant centers across Europe and the United States. 54 SR-aGVHD and 41 steroid-refractory cGVHD (SR-cGVHD) patients were given ruxolitinib. The overall response rate (ORR) was 81.5% in SR-aGVHD including 46.3% complete responses (CR) with a low rate of GVHD relapse of 6.8% (28). Impressive 6-month-survival of 79% (67.3–90.7%, 95% CI) was reported. In SR-cGVHD patients, an ORR of 85.4% was observed with a low rate of relapse (5.7%). The 6-month survival in this group was 97.4% (92.3%–100%, 95% CI). Regarding adverse events, cytomegalovirus (CMV) reactivation rate of 33.3% and 14.6% was noted in SR-aGVHD and SR-cGVHD patients respectively. A case of CMV-retinitis was reported, and all CMV cases were subsequently controlled by antiviral medication. Cytopenias were observed in 55.5% and 17% of SR-aGVHD and SR-cGVHD patients, respectively. Severe cytopenias (grades 3 and 4) were found in 33.3% and 7.3% SR-aGVHD and SR-cGVHD respectively. This was confounded by the presence of cytopenias preceding ruxolitinib therapy. A low malignancy relapse rate of 9.3% in SR-aGVHD and 2.4% SR-cGVHD patients was noted.

Itacitinib, which is an investigational tyrosine kinase inhibitor selective for JAK1, has been studied in aGVHD. Itacitinib was used in the first registered study of a JAK inhibitor in patients with acute GVHD (INCB 39110-108) where patients with steroid-naïve or steroid refractory aGVHD were randomized 1:1 to received either 200 mg (n=14) or 300 mg (n=15) daily dose (30, 67). In this phase I study, only 1 DLT was observed which was thrombocytopenia attributed to GVHD progression in a patient with pre-existing thrombocytopenia. The most common non-hematologic treatment emergent adverse event (TEAE) was diarrhea (48.3%) although 79% of those patients had GI GVHD at baseline. GI hemorrhage was reported in 3 and 2 patients at the 200 mg and 300 mg dose groups respectively. 1 patient had 2 CMV infections. Most commonly reported hematologic TEAEs were anemia (37.9%), decreased platelet count (27.6%), thrombocytopenia (24.1%). Grade 3-4 thrombocytopenia was reported in 2 and 3 patients on the 200 mg and 300 mg dose groups respectively. Sepsis was the most common infection AE occurring in 2 and 3 patients on the 200 mg and 300 mg dose groups respectively. Four patients, all in the 200 mg dose group, had CMV infection. The most common itacitinib-related TEAEs were anemia and decreased platelet counts which occurred more in the 300 mg dose group. Day 28 ORR in all patients for the 200 mg and 300 mg dose levels was 78.6% and 66.7% respectively. Day 28 ORR for steroid-naïve and steroid refractory patients were 75.0% and 70.6% respectively. Median duration of response was not reached for steroid-naïve aGVHD patients and 386 days for SR-aGVHD patients. In steroid-naïve aGVHD patients, 6- and 12- month OS was 75.0% and 58.3% respectively, whereas in the SR-aGVHD group, 6- and 12-month OS rates were 47.1% and 41.2% respectively.

Ruxolitinib was studied prospectively for the treatment of SR-aGVHD in an open-label phase II study (REACH1) (29). Ruxolitinib was given at a dose of 5 mg twice daily, with the possibility to increase to 10 mg twice daily in the absence of cytopenias. Ruxolitinib could be tapered after 6 months of therapy in patients who had discontinued corticosteroids for 8 weeks and had achieved a CR or very good partial remission (VGPR). Day 28 ORR, the primary endpoint of the study, was 54.9% (95% CI, 42.7%-66.8%), where 26.8% achieved a CR, 9.9% achieved a VGPR, and 18.3% a partial response (PR). When analyzed by GVHD grade, ORR of 82.6%, 41.2% and 42.9% were observed in patients with grade II, III, and IV SR-aGVHD, respectively. The median time to first response was 7.0 days (range, 6-49). Median duration of response at 6 months was 345 days. The 6- and 12-month overall survival (OS) rates were 51.0% and 42.6% respectively. Adverse events observed were in line with expectations for patients with SR-aGVHD being treated with ruxolitinib. Hematologic treatment-emergent adverse events (TEAE)s were frequent, with the most common hematological TEAEs being anemia (64.8%), thrombocytopenia (62.0%) and neutropenia (47.9%). There were 2 cases of thrombotic microangiopathy. Infections occurred in 80.3% of patients, with the most frequent being CMV, where rates of infection, viremia, and retinitis were (12.7%), (5.6%), and (1.4%), respectively. Fatal treatment-related TEAEs included sepsis and pulmonary hemorrhage (1 subject each). These findings have since led to the FDA approval of ruxolitinib for SR-aGVHD (68).

The REACH1 study was subsequently followed up by the REACH2 study, which was a multicenter, open-label, randomized phase III study comparing ruxolitinib to best available therapy (BAT) in SR-aGVHD. 154 patients received ruxolitinib and 155 were assigned to the control arm. 49 patients (32%) crossed over to the ruxolitinib arm on or after day 28. ORR at day 28 was significantly higher in the ruxolitinib arm (62% *vs* 39%, p<0.001). CR rates were also higher (34% *vs*. 19%). Responses were more durable (40% *vs* 22% at day 56) and incidence of loss of response at 6 months was lower with ruxolitinib (10% *vs* 39%). Response rates were highest in grade II disease, although the odds ratio for response with ruxolitinib was highest in patients with grade IV disease at baseline (53% *vs*. 23%; odds ratio, 3.76; 95% CI,1.24 to 11.38). Failure-free survival (FFS) and OS were also significantly longer in the ruxolitinib arm (5.0 *vs* 1.0 months and 11.1 *vs* 6.5 months, respectively). The most common adverse events in the treatment *vs*. control arm were thrombocytopenia (33% *vs* 18%), anemia (30% *vs*. 28%) and CMV infection (26% *vs*. 21%). Grade 3 infections up to day 28 were reported in 34 patients (22%) who received ruxolitinib and in 28 patients (19%) in the control arm. Median time to first infection of grade 3 severity was 0.8 months in the ruxolitinib arm and 0.7 in the control arm. At the data cutoff date, incidence of grade 3 or higher bleeding was 12% *vs* 7% in the ruxolitinib and control arms respectively. Severe adverse events (SAE)s by day 28 were reported in 38% of patients in the ruxolitinib arm and 34% in the control arm. These data confirm results from the REACH1 study and ruxolitinib is now standard of care for SR-aGVHD.

The REACH3 study evaluated ruxolitinib in SR-cGVHD and was presented at the 2020 annual meeting of the American Society of Hematology (32). It was an open-label, randomized phase III trial comparing ruxolitinb to BAT. A total of 329 pts were randomized, 165 received ruxolitinib and 164 received BAT. 61 patients (37.2%) crossed over the ruxolitinib arm. The primary endpoint was ORR at week 24. Ruxolitinib was superior to best available therapy with an ORR of 49.7% *vs* 25.6% (odds ratio, 2.99; P < 0.001) and the CR rate was higher with ruxolitinib as well (6.7% *vs* 3.0%). Key secondary endpoints also showed superiority of ruxolitinib, where FFS was improved in the ruxolitinib group (median FFS, >18.6 *vs* 5.7 months; HR, 0.37 [95% CI, 0.27-0.51]; P < 0.001), and improved response rate on the modified Lee symptom score (defined as a 7 point or greater reduction in symptom score) (24.2% *vs* 11.0%; odds ratio, 2.62; P = 0.001). Rates of AEs were comparable in both arms. The most common AEs of grade 3 or higher in both arms (ruxolitinib *vs* best available therapy) included thrombocytopenia (15.2% *vs* 10.1%), anemia (12.7% *vs* 7.6%), neutropenia (8.5% *vs* 3.8%) and pneumonia (8.5% *vs* 9.5%). Infections of any type occurred in 63.6% of ruxolitinib treated patients and 56.3% of best available therapy patients. FDA approval of ruxolitinib for SR-cGVHD is anticipated.

Baricitinib, another JAK1/2 inhibitor approved for rheumatoid arthritis, was used in a phase I/II study in patients with SR-cGVHD (69). No DLT was observed with the 2 mg dose of baricitinib. Possibly treatment-related AEs included upper respiratory infection in 13 patients, neutropenia in 6, hypophosphatemia in 12, and hypertriglyceridemia in 5. Notable viral reactivation included 6 patients with CMV, 7 patients with Epstein-Barr virus (EBV) and 5 patients with BK viruria; none of which required treatment. One patient was diagnosed with post-transplant lymphoproliferative disorder (PTLD) within 1 cycle on therapy who had EBV viremias and lymphadenopathy at enrollment. 11 SAEs were reported, of which 5 were possibly drug-related, and there were no deaths on study. ORR at 6 months was 63% and ORR at any time reached 90%. 1- and 2-year FFS was 74% and 37%, respectively.

Several published studies also support these findings, where ruxolitinib was given to refractory GVHD patients and are summarized in **Table 1**. These includes reports in adults (71–74, 76, 77, 79, 82) and pediatric patients (70, 75, 77–82), SR-aGVHD (70, 71, 73, 75, 77–81) as well as SR-cGVHD (71–82).

**Table 1:** Clinical studies of JAK inhibitors for the treatment of refractory GVHD.

Reference	Agent	Target	Study Type	Indication	N	Response	Survival
Spoerl et al. (38)	Ruxolitinib	JAK1/2	Pilot	SR- aGVHD	6	100%	NA
Zeiser et al. (28)	Ruxolitinib	JAK1/2	Retrospective	SR- aGVHD and SR-cGVHD	95	SR-aGVHD 81.5% SR-cGVHD 85.4%	6-mo SR-aGVHD 79% 6-mo SR-cGVHD 97.4%
Khandelwal et al. (70)	Ruxolitinib	JAK1/2	Retrospective	SR-aGVHD	13	45% (n=11)	7/13 alive at median follow up of 401 days
Maldonado et al. (71)	Ruxolitinib	JAK1/2	Retrospective	SR- aGVHD and SR-cGVHD	3 5	Overall ORR 85%	NA
Khoury et al. (72)	Ruxolitinib	JAK1/2	Retrospective	Steroid dependent-cGVHD	19	100%	NA
Abedin et al. (73)	Ruxolitinib	JAK1/2	Retrospective	SR- aGVHD and SR-cGVHD	19 24	84% 83%	6-mo FFS SR-aGVHD 58% SR-cGVHD 88%
Ferreira et al. (74)	Ruxolitinib	JAK1/2	Retrospective	SR-cGVHD	20	75%	11-mo 67%
Gonzalez Vicent et al. (75)	Ruxolitinib	JAK1/2	Retrospective	SR- aGVHD and SR-cGVHD	13 9	77% 89%	6-mo OS SR-aGVHD 30 +/-15% SR-cGVHD 100%
Modi et al. (76)	Ruxolitinib	JAK1/2	Retrospective	SR-cGVHD	46	1-yr 58%	1-yr FFS of 54.2%
Escamilla Gomez et al. (77)	Ruxolitinib	JAK1/2	Retrospective	SR- aGVHD and SR-cGVHD	23 56	69.5% 57.1%	6-mo SR-aGVHD 47% 1-yr SR-cGVHD 81%
Uygun et al. (78)	Ruxolitinib	JAK1/2	Retrospective	SR- aGVHD, overlap syndrome and SR-cGVHD	13 1 15	84.6% 100% 80.0%	90% alive at end of study
Dang et al. (79)	Ruxolitinib	JAK1/2	Retrospective	SR- aGVHD and SR-cGVHD	10 28	100% 82.1%	NA
Jagasia et al. (29)	Ruxolitinib	JAK1/2	Phase II	SR-aGVHD	71	Day 28 ORR 54.9%	6-mo 51.0%
Zeiser et al. (31)	Ruxolitinib	JAK1/2	Phase III	SR-aGVHD	309 (ruxolitinib 154)	Day 28 ORR 62%	6-mo 59.5% *vs* 50.3%
Shroeder et al. (30)	Itacitinib	JAK1	Phase 1	Steroid-naïve aGVHD and SR-aGVHD	12 17	Day 28 ORR 75.0% 70.6%	6-mo 75.0% 47.1%
Zeiser et al. (32)	Ruxolitinib	JAK1/2	Phase III	SR-cGVHD	329 (ruxolitinib 165)	Week 24 ORR 49.7% *vs* 25.6%	FFS >18.6 mo *vs* 5.7 mo
Holtzman et al. (69)	Baricitinib	JAK1/2	Phase I/II	SR-cGVHD	20	63%	1-yr FFS 74%
Yang et al. (80)	Ruxolitinib	JAK1/2	Retrospective	SR- aGVHD and SR-cGVHD	17 36	64.7% 80.6%	6-mo SR-aGVHD 92.3% SR-cGVHD 100%
Mozo et al. (81)	Ruxolitinib	JAK1/2	Retrospective	SR- aGVHD and SR-cGVHD	8 12 (1 patient treated for both acute and chronic)	87% 91%	1-yr SR-aGVHD 64.8%% 2-yr SR-cGVHD 76.4%
Wang et al. (82)	Ruxolitinib	JAK1/2	Retrospective	SR-cGVHD	20	70%	90% alive at end of study

NA, not applicable.

### Studies in Upfront GVHD Therapy

Ruxolitinib has been used in combination with corticosteroids as upfront treatment for aGVHD in a prospective study of patients receiving haploidentical transplants (83). 32 patients were treated, and day 28 CR rate was 96.9%. Response rates were significantly higher than those observed in a group of matched historical controls treated with corticosteroids alone. cGVHD rates were low with a 1-year and 2-year cumulative incidence rates of 9.4% and 13.8%, respectively. Estimated 1-year OS was 73.4%. aGVHD recurred in 31.2% of patients, mostly in the setting of taper of immunosuppressive medications. Ruxolitinib dose was initially 5 mg twice daily, but later reduced in the study protocol for patients receiving azoles due to a high incidence of cytopenias. CMV reactivation was seen in 78.1% of patients, with 2 cases of CMV encephalitis, one of them proved fatal. EBV viremia was detected in 87.5% of patients, and 2 patients developed PTLD. Other notable infections included a case of pulmonary aspergillosis and a case of Pneumocystis jirovecii pneumonia, both successfully treated. Grade 3-4 thrombocytopenia occurred in 3 patients, all before the protocol-recommended dose reduction of ruxolitinib. Subsequent patients developed reversible thrombocytopenia that did not require a dose reduction. No neutropenia was observed and 2 cases of thrombotic microangiopathy were observed that resolved after reduction of calcineurin inhibitor.

GRAVITAS-301 was a placebo-controlled, randomized, phase III study of corticosteroids with or without itacitinib as upfront treatment for aGVHD (84). Randomization was 1:1 where 219 patients received itacitinib and 220 received placebo. The study failed to meet its primary endpoint, which was a statistically significant improvement of the day 28 ORR (itacitinib *vs* placebo, 74% *vs* 66%, p=0.08). Post-hoc analysis however of the day 28 CR rates showed a significant improvement for itacitinib *vs* placebo when stratified by aGVHD risk status (odds ratio, 1.66; 95% CI, 1.14–2.44; P=0.008). Median time to first response was 8 days in both groups. Median duration of response was also similar. Notably, the 6-month estimates of non-relapse mortality were similar in both groups (itacitinib *vs* placebo, 18% *vs* 19%). At median follow-up of 267 days, the 1-year OS estimated with 70% for itacitinib and 66% for placebo. Treatment-related AEs were also similar in both groups.

### Studies in GVHD Prophylaxis

Majority of the preclinical data in mouse models described above studied the ability of JAK inhibition to prevent GVHD, whereas clinical studies focused on treating refractory disease, which is in line with the clinical development of agents for novel indications. Studies using JAK inhibitors in the prophylaxis setting are emerging, however. One study of 12 patients with myelofibrosis continued ruxolitinib therapy until stable engraftment (85). Only 1 case of aGVHD was reported before day +100, however 4 patients developed aGVHD after taper of cyclosporine. All patients were alive at the time of analysis. CMV reactivation occurred in 5 patients, 1 of whom developed CMV colitis. All responded to treatment with ganciclovir. 2 patients discontinued therapy due to cytopenias. A reduction in levels of inflammatory cytokines was reported as well. Another study administered ruxolitinib to calcineurin inhibitor intolerant patients as aGVHD prophylaxis (86). 10 patients were enrolled into this pilot study. After ruxolitinib initiation, only 1 patient developed grade II skin aGVHD, and 1 patient developed severe aGVHD after day +100. 2 patients developed cGVHD after ruxolitinib taper. CMV reactivation was reported in 4 patients, and EBV viremia was reported in 3 patients. None developed CMV disease or PTLD. Finally, a study employed post-transplant cyclophosphamide with ruxolitinib as a calcineurin-free GVHD prophylaxis regimen (87). 20 patients with primary or secondary myelofibrosis were enrolled. 1 patient experienced primary graft failure and 2 patients died before engraftment. Dose reduction in ruxolitinib was required in 11 patients due to severe poor graft function. Overall, the regimen was well tolerated with 30% grade 3-4 non-hematolgic toxicity, 45% viral reactivation rate, and severe sepsis reported in 15% of patients. Incidence of grade II–IV aGVHD was 25%, grade III-IV aGVHD was 15%. No severe cGVHD cases were reported, and moderate cGVHD occurred in 20% of patients. Only 2 patients required systemic steroids. The 2-year OS and event-free survival were 85% and 72% respectively.

The GRAVITAS-119 trial is a single arm phase I study of itacitinib in combination with calcineurin inhibitor based interventions for the prophylaxis of GVHD (88). The primary endpoint was day 28 hematologic recovery. 65 patients were enrolled, all patients achieved hematologic recovery which included 1 patient with myelofibrosis who achieved neutrophil engraftment by day 31. 2 patients developed secondary graft failure. In 63 evaluable patients, cumulative incidence of grade III-IV aGVHD was 4.8% and 1 year GVHD-relapse-free survival (GRFS) was 38.5%. The addition of itacitinib was well tolerated; the most common grade 3-4 hematologic AEs included thrombocytopenia (49%) and anemia (31%). CMV reactivation occurred in 26% of patients, and 12% had EBV infection. No cases of PTLD were reported. 1 patient developed invasive bronchopulmonary aspergillosis. The most common reasons for itacitinib discontinuation were AEs (22%) and relapse (17%). 15 patients in the per-protocol population died, 2 of which were due to infections and 1 due to intracranial hemorrhage. Other ongoing studies for GVHD prophylaxis using itacitinib include a phase I study in patients receiving haploidentical transplants (NCT03755414, www.ClinicalTrials.gov), and a phase IIa study of patients receiving reduced-intensity conditioning (NCT04339101 www.ClinicalTrials.gov). A phase I study with baricitinib for GVHD prophylaxis is ongoing as well (NCT04131738, www.ClinicalTrials.gov).

## Conclusions

JAK inhibitors are well positioned as therapies for complications common after cellular therapies such as GVHD in the setting of HSCT, and CRS in the setting of IECT. The JAK/STAT pathway is involved in the signaling of several cytokines that are critical to the pathogenesis of GVHD and CRS as described above. JAK inhibition has been shown to ameliorate the pathogenic T cell and macrophage proliferation and activation in experimental models and enhance Treg function and proliferation, results which have now been translated to successful clinical studies in refractory GVHD. Most importantly, JAK inhibition does not seem to interfere with the graft *versus* leukemia effect or the activity of CART cells used in IECT which is a common concern with the blunting of immune activity (36, 40, 65). Results from further studies in the upfront or prophylactic setting are highly anticipated, despite the negative results from the GRAVITAS-301 study (84).

Despite the success of ruxolitinib in the treatment of SR-aGVHD and SR-cGVHD, adverse events remain common and the response rates are far from perfect. Other JAK inhibitors may prove more efficacious or less toxic especially as they may differ in the off-target effects. Combination therapies with agents that target other pathways such as CD28:CD80/86 constimulation with abatacept (89), Rho-associated kinase 2 with belumosudil (90), or CSF-1R blockade with axatilimab (91) may also prove beneficial as we refine our understanding of the pathogenic pathways controlling development of GVHD.

## Author Contributions

AA wrote the manuscript. MM wrote and edited the manuscript. All authors contributed to the article and approved the submitted version.

## Funding

MM was supported by NIBIB R01EB025221.

## Conflict of Interest

AA received research funding and advisory board fees from Incyte Corporation. MM received consulting fees from Ossium Health.

## Publisher’s Note

All claims expressed in this article are solely those of the authors and do not necessarily represent those of their affiliated organizations, or those of the publisher, the editors and the reviewers. Any product that may be evaluated in this article, or claim that may be made by its manufacturer, is not guaranteed or endorsed by the publisher.

## References

[1] Kanate AS Majhail NS Savani BN Bredeson C Champlin RE Crawford S. Indications for Hematopoietic Cell Transplantation and Immune Effector Cell Therapy: Guidelines From the American Society for Transplantation and Cellular Therapy. Biol Blood Marrow Transplant (2020) 26:1247–56. 10.1016/j.bbmt.2020.03.002 32165328

[2] D'Souza A Zhu X. Current Uses and Outcomes of Hematopoietic Cell Transplantation (HCT): CIBMTR Summary Slides. (2016). https://www.cibmtr.org/ReferenceCenter/SlidesReports/SummarySlides/pages/index.aspx

[3] Jagasia MH Greinix HT Arora M Williams KM Wolff D Cowen EW. National Institutes of Health Consensus Development Project on Criteria for Clinical Trials in Chronic Graft-*Versus*-Host Disease: I. The 2014 Diagnosis and Staging Working Group Report. Biol Blood Marrow Transplant (2015) 21:389–401.e1. 10.1016/j.bbmt.2015.02.025 25529383 PMC4329079

[4] Filipovich AH Weisdorf D Pavletic S Socie G Wingard JR Lee SJ. National Institutes of Health Consensus Development Project on Criteria for Clinical Trials in Chronic Graft-*Versus*-Host Disease: I. Diagnosis and Staging Working Group Report. Biol Blood Marrow Transplant (2005) 11:945–56. 10.1016/j.bbmt.2005.09.004 16338616

[5] Zeiser R Blazar BR. Acute Graft-*Versus*-Host Disease - Biologic Process, Prevention, and Therapy. N Engl J Med (2017) 377:2167–79. 10.1056/NEJMra1609337 PMC6034180 29171820

[6] Zeiser R Blazar BR. Pathophysiology of Chronic Graft-*Versus*-Host Disease and Therapeutic Targets. N Engl J Med (2017) 377:2565–79. 10.1056/NEJMra1703472 29281578

[7] Jagasia M Arora M Flowers ME Chao NJ McCarthy PL Cutler CS. Risk Factors for Acute GVHD and Survival After Hematopoietic Cell Transplantation. Blood (2012) 119:296–307. 10.1182/blood-2011-06-364265 22010102 PMC3251233

[8] Flowers ME Inamoto Y Carpenter PA Lee SJ Kiem HP Petersdorf EW. Comparative Analysis of Risk Factors for Acute Graft-*Versus*-Host Disease and for Chronic Graft-*Versus*-Host Disease According to National Institutes of Health Consensus Criteria. Blood (2011) 117:3214–9. 10.1182/blood-2010-08-302109 PMC3062319 21263156

[9] Martin PJ Rizzo JD Wingard JR Ballen K Curtin PT Cutler C. First- and Second-Line Systemic Treatment of Acute Graft-*Versus*-Host Disease: Recommendations of the American Society of Blood and Marrow Transplantation. Biol Blood Marrow Transplant (2012) 18:1150–63. 10.1016/j.bbmt.2012.04.005 PMC3404151 22510384

[10] Carpenter PA Logan BR Lee SJ Weisdorf DJ Johnston L Costa LJ. A Phase II/III Randomized, Multicenter Trial of Prednisone/Sirolimus *Versus* Prednisone/ Sirolimus/Calcineurin Inhibitor for the Treatment of Chronic Graft-Versus-Host Disease: BMT CTN 0801. Haematologica (2018) 103:1915–24. 10.3324/haematol.2018.195123 PMC6278959 29954931

[11] Penack O Marchetti M Ruutu T Aljurf M Bacigalupo A Bonifazi F. Prophylaxis and Management of Graft *Versus* Host Disease After Stem-Cell Transplantation for Haematological Malignancies: Updated Consensus Recommendations of the European Society for Blood and Marrow Transplantation. Lancet Haematol (2020) 7:e157–67. 10.1016/S2352-3026(19)30256-X 32004485

[12] Harris AC Young R Devine S Hogan WJ Ayuk F Bunworasate U. International, Multicenter Standardization of Acute Graft-*Versus*-Host Disease Clinical Data Collection: A Report From the Mount Sinai Acute GVHD International Consortium. Biol Blood Marrow Transplant (2016) 22:4–10. 10.1016/j.bbmt.2015.09.001 26386318 PMC4706482

[13] MacMillan ML Robin M Harris AC DeFor TE Martin PJ Alousi A. A Refined Risk Score for Acute Graft-*Versus*-Host Disease That Predicts Response to Initial Therapy, Survival, and Transplant-Related Mortality. Biol Blood Marrow Transplant (2015) 21:761–7. 10.1016/j.bbmt.2015.01.001 PMC4359643 25585275

[14] MacMillan ML DeFor TE Weisdorf DJ. The Best Endpoint for Acute GVHD Treatment Trials. Blood (2010) 115:5412–7. 10.1182/blood-2009-12-258442 20388871

[15] Lee SJ Nguyen TD Onstad L Bar M Krakow EF Salit RB. Success of Immunosuppressive Treatments in Patients With Chronic Graft-*Versus*-Host Disease. Biol Blood Marrow Transplant (2018) 24:555–62. 10.1016/j.bbmt.2017.10.042 PMC5826880 29133250

[16] Chou CK Turtle CJ. Assessment and Management of Cytokine Release Syndrome and Neurotoxicity Following CD19 CAR-T Cell Therapy. Expert Opin Biol Ther (2020) 20:653–64. 10.1080/14712598.2020.1729735 PMC7393694 32067497

[17] Freyer CW Porter DL. Cytokine Release Syndrome and Neurotoxicity Following CAR T-Cell Therapy for Hematologic Malignancies. J Allergy Clin Immunol (2020) 146:940–8. 10.1016/j.jaci.2020.07.025 32771558

[18] Maude SL Laetsch TW Buechner J Rives S Boyer M Bittencourt H. Tisagenlecleucel in Children and Young Adults With B-Cell Lymphoblastic Leukemia. N Engl J Med (2018) 378:439–48. 10.1056/NEJMoa1709866 PMC5996391 29385370

[19] Schuster SJ Bishop MR Tam CS Waller EK Borchmann P McGuirk JP. Tisagenlecleucel in Adult Relapsed or Refractory Diffuse Large B-Cell Lymphoma. N Engl J Med (2019) 380:45–56. 10.1056/NEJMoa1804980 30501490

[20] Neelapu SS Locke FL Bartlett NL Lekakis LJ Miklos DB Jacobson CA. Axicabtagene Ciloleucel CAR T-Cell Therapy in Refractory Large B-Cell Lymphoma. N Engl J Med (2017) 377:2531–44. 10.1056/NEJMoa1707447 PMC5882485 29226797

[21] Lee DW Santomasso BD Locke FL Ghobadi A Turtle CJ Brudno JN. ASTCT Consensus Grading for Cytokine Release Syndrome and Neurologic Toxicity Associated With Immune Effector Cells. Biol Blood Marrow Transplant (2019) 25:625–38. 10.1016/j.bbmt.2018.12.758 PMC12180426 30592986

[22] Yanez L Alarcon A Sanchez-Escamilla M Perales MA. How I Treat Adverse Effects of CAR-T Cell Therapy. ESMO Open (2020) 4:e000746. 32839196 10.1136/esmoopen-2020-000746 PMC7451454

[23] Alexander M Culos K Roddy J Shaw R Bachmeier C Shigle TL. Chimeric Antigen Receptor T Cell Therapy: A Comprehensive Review of Clinical Efficacy, Toxicity, and Best Practices for Outpatient Administration. Transplant Cell Ther (2021) 27:558–70. 10.1016/j.jtct.2021.01.014 33910041

[24] Leng C Gries M Ziegler J Lokshin A Mascagni P Lentzsch S. Reduction of Graft-*Versus*-Host Disease by Histone Deacetylase Inhibitor Suberonylanilide Hydroxamic Acid Is Associated With Modulation of Inflammatory Cytokine Milieu and Involves Inhibition of STAT1. Exp Hematol (2006) 34:776–87. 10.1016/j.exphem.2006.02.014 16728283

[25] Ma HH Ziegler J Li C Sepulveda A Bedeir A Grandis J. Sequential Activation of Inflammatory Signaling Pathways During Graft-*Versus*-Host Disease (GVHD): Early Role for STAT1 and STAT3. Cell Immunol (2011) 268:37–46. 10.1016/j.cellimm.2011.01.008 21376308 PMC3061826

[26] Ma H Lu C Ziegler J Liu A Sepulveda A Okada H. Absence of Stat1 in Donor CD4(+) T Cells Promotes the Expansion of Tregs and Reduces Graft-*Versus*-Host Disease in Mice. J Clin Invest (2011) 121:2554–69. 10.1172/JCI43706 PMC3223821 21670504

[27] Capitini CM Nasholm NM Chien CD Larabee SM Qin H Song YK. Absence of STAT1 in Donor-Derived Plasmacytoid Dendritic Cells Results in Increased STAT3 and Attenuates Murine GVHD. Blood (2014) 124:1976–86. 10.1182/blood-2013-05-500876 PMC4168352 25079358

[28] Zeiser R Burchert A Lengerke C Verbeek M Maas-Bauer K Metzelder SK. Ruxolitinib in Corticosteroid-Refractory Graft-*Versus*-Host Disease After Allogeneic Stem Cell Transplantation: A Multicenter Survey. Leukemia (2015) 29:2062–8. 10.1038/leu.2015.212 PMC4854652 26228813

[29] Jagasia M Perales MA Schroeder MA Ali H Shah NN Chen YB. Ruxolitinib for the Treatment of Steroid-Refractory Acute GVHD (REACH1): A Multicenter, Open-Label Phase 2 Trial. Blood (2020) 135:1739–49. 10.1182/blood.2020004823 PMC7229262 32160294

[30] Schroeder MA Khoury HJ Jagasia M Ali H Schiller GJ Staser K. A Phase 1 Trial of Itacitinib, a Selective JAK1 Inhibitor, in Patients With Acute Graft-*Versus*-Host Disease. Blood Adv (2020) 4:1656–69. 10.1182/bloodadvances.2019001043 PMC7189299 32324888

[31] Zeiser R von Bubnoff N Butler J Mohty M Niederwieser D Or R. Ruxolitinib for Glucocorticoid-Refractory Acute Graft-*Versus*-Host Disease. N Engl J Med (2020) 382:1800–10. 10.1056/NEJMoa1917635 32320566

[32] Zeiser R Polverelli N Ram R Hashmi SK Chakraverty R Middeke JM. Ruxolitinib for Glucocorticoid-Refractory Chronic Graft-*Versus*-Host Disease. N Engl J Med (2021) 385:228–38. 10.1056/NEJMoa2033122 34260836

[33] Murray PJ. The JAK-STAT Signaling Pathway: Input and Output Integration. J Immunol (2007) 178:2623–9. 10.4049/jimmunol.178.5.2623 17312100

[34] Park JH Frigault MJ Maziarz RT Naim A Burke L Tian C. Trial in Progress: A Phase 2, Single-Arm, Open-Label Study of Itacitinib (ITA) for the Prevention of Chimeric Antigen Receptor (CAR) T-Cell–Induced Cytokine Release Syndrome (CRS). Biol Blood Marrow Transplant (2020) 26:S269. 10.1016/j.bbmt.2019.12.436

[35] Sugerman PB Faber SB Willis LM Petrovic A Murphy GF Pappo J. Kinetics of Gene Expression in Murine Cutaneous Graft-*Versus*-Host Disease. Am J Pathol (2004) 164:2189–202. 10.1016/S0002-9440(10)63776-5 PMC1615752 15161652

[36] Choi J Cooper ML Alahmari B Ritchey J Collins L Holt M. Pharmacologic Blockade of JAK1/JAK2 Reduces GvHD and Preserves the Graft-*Versus*-Leukemia Effect. PloS One (2014) 9:e109799. 10.1371/journal.pone.0109799 25289677 PMC4188578

[37] Choi J Ziga ED Ritchey J Collins L Prior JL Cooper ML. IFNgammaR Signaling Mediates Alloreactive T-Cell Trafficking and GVHD. Blood (2012) 120:4093–103. 10.1182/blood-2012-01-403196 PMC3496960 22972985

[38] Spoerl S Mathew NR Bscheider M Schmitt-Graeff A Chen S Mueller T. Activity of Therapeutic JAK 1/2 Blockade in Graft-*Versus*-Host Disease. Blood (2014) 123:3832–42. 10.1182/blood-2013-12-543736 24711661

[39] Zhang Y Joe G Hexner E Zhu J Emerson SG. Alloreactive Memory T Cells are Responsible for the Persistence of Graft-*Versus*-Host Disease. J Immunol (2005) 174:3051–8. 10.4049/jimmunol.174.5.3051 15728519

[40] Choi J Cooper ML Staser K Ashami K Vij KR Wang B. Baricitinib-Induced Blockade of Interferon Gamma Receptor and Interleukin-6 Receptor for the Prevention and Treatment of Graft-*Versus*-Host Disease. Leukemia (2018) 32:2483–94. 10.1038/s41375-018-0123-z PMC6168427 29691471

[41] Carniti C Gimondi S Vendramin A Recordati C Confalonieri D Bermema A. Pharmacologic Inhibition of JAK1/JAK2 Signaling Reduces Experimental Murine Acute GVHD While Preserving GVT Effects. Clin Cancer Res (2015) 21:3740–9. 10.1158/1078-0432.CCR-14-2758 25977345

[42] Yarilina A Xu K Chan C Ivashkiv LB. Regulation of Inflammatory Responses in Tumor Necrosis Factor-Activated and Rheumatoid Arthritis Synovial Macrophages by JAK Inhibitors. Arthritis Rheum (2012) 64:3856–66. 10.1002/art.37691 PMC3510320 22941906

[43] Heine A Held SA Daecke SN Wallner S Yajnanarayana SP Kurts C. The JAK-Inhibitor Ruxolitinib Impairs Dendritic Cell Function *In Vitro* and *In Vivo* . Blood (2013) 122:1192–202. 10.1182/blood-2013-03-484642 23770777

[44] Stickel N Hanke K Marschner D Prinz G Kohler M Melchinger W. MicroRNA-146a Reduces MHC-II Expression via Targeting JAK/STAT Signaling in Dendritic Cells After Stem Cell Transplantation. Leukemia (2017) 31:2732–41. 10.1038/leu.2017.137 PMC6231537 28484267

[45] Hulsdunker J Ottmuller KJ Neeff HP Koyama M Gao Z Thomas OS. Neutrophils Provide Cellular Communication Between Ileum and Mesenteric Lymph Nodes at Graft-*Versus*-Host Disease Onset. Blood (2018) 131:1858–69. 10.1182/blood-2017-10-812891 PMC5909763 29463561

[46] Betts BC Bastian D Iamsawat S Nguyen H Heinrichs JL Wu Y. Targeting JAK2 Reduces GVHD and Xenograft Rejection Through Regulation of T Cell Differentiation. Proc Natl Acad Sci USA (2018) 115:1582–7. 10.1073/pnas.1712452115 PMC5816153 29382747

[47] Zorn E Kim HT Lee SJ Floyd BH Litsa D Arumugarajah S. Reduced Frequency of FOXP3+ CD4+CD25+ Regulatory T Cells in Patients With Chronic Graft-*Versus*-Host Disease. Blood (2005) 106:2903–11. 10.1182/blood-2005-03-1257 PMC1895303 15972448

[48] Koreth J Matsuoka K Kim HT McDonough SM Bindra B Alyea EP 3rd. Interleukin-2 and Regulatory T Cells in Graft-*Versus*-Host Disease. N Engl J Med (2011) 365:2055–66. 10.1056/NEJMoa1108188 PMC3727432 22129252

[49] Forcade E Kim HT Cutler C Wang K Alho AC Nikiforow S. Circulating T Follicular Helper Cells With Increased Function During Chronic Graft-*Versus*-Host Disease. Blood (2016) 127:2489–97. 10.1182/blood-2015-12-688895 PMC4874229 26944544

[50] Alexander KA Flynn R Lineburg KE Kuns RD Teal BE Olver SD. CSF-1-Dependant Donor-Derived Macrophages Mediate Chronic Graft-*Versus*-Host Disease. J Clin Invest (2014) 124:4266–80. 10.1172/JCI75935 PMC4191032 25157821

[51] Ryu DB Lim JY Kim TW Shin S Lee SE Park G. Preclinical Evaluation of JAK1/2 Inhibition by Ruxolitinib in a Murine Model of Chronic Graft-*Versus*-Host Disease. Exp Hematol (2021) 98:36–46.e2. 10.1016/j.exphem.2021.03.004 33811972

[52] Teachey DT Lacey SF Shaw PA Melenhorst JJ Maude SL Frey N. Identification of Predictive Biomarkers for Cytokine Release Syndrome After Chimeric Antigen Receptor T-Cell Therapy for Acute Lymphoblastic Leukemia. Cancer Discovery (2016) 6:664–79. 10.1158/2159-8290.CD-16-0040 PMC5448406 27076371

[53] Singh N Hofmann TJ Gershenson Z Levine BL Grupp SA Teachey DT. Monocyte Lineage-Derived IL-6 Does Not Affect Chimeric Antigen Receptor T-Cell Function. Cytotherapy (2017) 19:867–80. 10.1016/j.jcyt.2017.04.001 PMC6676485 28506444

[54] Norelli M Camisa B Barbiera G Falcone L Purevdorj A Genua M. Monocyte-Derived IL-1 and IL-6 Are Differentially Required for Cytokine-Release Syndrome and Neurotoxicity Due to CAR T Cells. Nat Med (2018) 24:739–48. 10.1038/s41591-018-0036-4 29808007

[55] Giavridis T van der Stegen SJC Eyquem J Hamieh M Piersigilli A Sadelain M. CAR T Cell-Induced Cytokine Release Syndrome Is Mediated by Macrophages and Abated by IL-1 Blockade. Nat Med (2018) 24:731–8. 10.1038/s41591-018-0041-7 PMC6410714 29808005

[56] Strati P Ahmed S Kebriaei P Nastoupil LJ Claussen CM Watson G. Clinical Efficacy of Anakinra to Mitigate CAR T-Cell Therapy-Associated Toxicity in Large B-Cell Lymphoma. Blood Adv (2020) 4:3123–7. 10.1182/bloodadvances.2020002328 PMC7362369 32645136

[57] Maschalidi S Sepulveda FE Garrigue A Fischer A de Saint Basile G. Therapeutic Effect of JAK1/2 Blockade on the Manifestations of Hemophagocytic Lymphohistiocytosis in Mice. Blood (2016) 128:60–71. 10.1182/blood-2016-02-700013 27222478

[58] Albeituni S Verbist KC Tedrick PE Tillman H Picarsic J Bassett R. Mechanisms of Action of Ruxolitinib in Murine Models of Hemophagocytic Lymphohistiocytosis. Blood (2019) 134:147–59. 10.1182/blood.2019000761 PMC6624972 31015190

[59] Huarte E Peel MT Verbist K Fay BL Bassett R Albeituni S. Ruxolitinib, a JAK1/2 Inhibitor, Ameliorates Cytokine Storm in Experimental Models of Hyperinflammation Syndrome. Front Pharmacol (2021) 12:650295. 10.3389/fphar.2021.650295 33981229 PMC8107823

[60] Meyer LK Verbist KC Albeituni S Scull BP Bassett RC Stroh AN. JAK/STAT Pathway Inhibition Sensitizes CD8 T Cells to Dexamethasone-Induced Apoptosis in Hyperinflammation. Blood (2020) 136:657–68. 10.1182/blood.2020006075 PMC7414590 32530039

[61] Hansen S Alduaij W Biggs CM Belga S Luecke K Merkeley H. Ruxolitinib as Adjunctive Therapy for Secondary Hemophagocytic Lymphohistiocytosis: A Case Series. Eur J Haematol (2021) 106:654–61. 10.1111/ejh.13593 33523540

[62] Ahmed A Merrill SA Alsawah F Bockenstedt P Campagnaro E Devata S. Ruxolitinib in Adult Patients With Secondary Haemophagocytic Lymphohistiocytosis: An Open-Label, Single-Centre, Pilot Trial. Lancet Haematol (2019) 6:e630–7. 10.1016/S2352-3026(19)30156-5 PMC8054981 31537486

[63] Sin JH Zangardi ML. Ruxolitinib for Secondary Hemophagocytic Lymphohistiocytosis: First Case Report. Hematol Oncol Stem Cell Ther (2019) 12:166–70. 10.1016/j.hemonc.2017.07.002 28834694

[64] Trantham T Auten J Muluneh B Van Deventer H. Ruxolitinib for the Treatment of Lymphoma-Associated Hemophagocytic Lymphohistiocytosis: A Cautionary Tale. J Oncol Pharm Pract (2020) 26:1005–8. 10.1177/1078155219878774 31575356

[65] Huarte E O'Connor RS Peel MT Nunez-Cruz S Leferovich J Juvekar A. Itacitinib (INCB039110), a JAK1 Inhibitor, Reduces Cytokines Associated With Cytokine Release Syndrome Induced by CAR T-Cell Therapy. Clin Cancer Res (2020) 26:6299–309. 10.1158/1078-0432.CCR-20-1739 PMC7895329 32998963

[66] Uy GL Rettig MP Christ S Aldoss I Byrne MT Erba HP. Prophylactic Ruxolitinib for Cytokine Release Syndrome (CRS) in Relapse/Refractory (R/R) AML Patients Treated With Flotetuzumab. Blood (2020) 136:19–21. 10.1182/blood-2020-134612

[67] Schroeder MA Khoury HJ Jagasia M Ali H Schiller GJ Arbushites M. A Phase I Trial of Janus Kinase (JAK) Inhibition With INCB039110 in Acute Graft-*Versus*-Host Disease (aGVHD). Blood (2016) 128:390–0. 10.1182/blood.V128.22.390.390

[68] Abedin SM Hamadani M. Ruxolitinib: A Potential Treatment for Corticosteroid Refractory Acute Graft-*Versus*-Host Disease. Expert Opin Investig Drugs (2020) 29:423–7. 10.1080/13543784.2020.1757069 32293938

[69] Holtzman NG Im A Ostojic A Curtis LM Parsons-Wandell L Nashed J. Efficacy and Safety of Baricitinib in Refractory Chronic Graft-*Versus*-Host Disease (cGVHD): Preliminary Analysis Results of a Phase 1/2 Study. Blood (2020) 136:1–1. 10.1182/blood-2020-140392 32430499

[70] Khandelwal P Teusink-Cross A Davies SM Nelson AS Dandoy CE El-Bietar J. Ruxolitinib as Salvage Therapy in Steroid-Refractory Acute Graft-*Versus*-Host Disease in Pediatric Hematopoietic Stem Cell Transplant Patients. Biol Blood Marrow Transplant (2017) 23:1122–7. 10.1016/j.bbmt.2017.03.029 28344057

[71] Sarmiento Maldonado M Ramirez Villanueva P Bertin Cortes-Monroy P Jara Arias V Soto Donoso K Uribe Gonzalez P. Compassionate Use of Ruxolitinib in Acute and Chronic Graft *Versus* Host Disease Refractory Both to Corticosteroids and Extracorporeal Photopheresis. Exp Hematol Oncol (2017) 6:32. 10.1186/s40164-017-0092-3 29214116 PMC5712115

[72] Khoury HJ Langston AA Kota VK Wilkinson JA Pusic I Jillella A. Ruxolitinib: A Steroid Sparing Agent in Chronic Graft-*Versus*-Host Disease. Bone Marrow Transplant (2018) 53:826–31. 10.1038/s41409-017-0081-5 PMC6041160 29367708

[73] Abedin S McKenna E Chhabra S Pasquini M Shah NN Jerkins J. Efficacy, Toxicity, and Infectious Complications in Ruxolitinib-Treated Patients With Corticosteroid-Refractory Graft-*Versus*-Host Disease After Hematopoietic Cell Transplantation. Biol Blood Marrow Transplant (2019) 25:1689–94. 10.1016/j.bbmt.2019.04.003 30965140

[74] Ferreira AM Pontes da Silva CA Pereira AD Szor RS Medeiros da Fonseca ARB Serpa MG. Ruxolitinib in Steroid-Refractory Chronic Graft-*Versus*-Host Disease: Experience of a Single Center. Bone Marrow Transplant (2018) 53:503–6. 10.1038/s41409-017-0068-2 29330403

[75] Gonzalez Vicent M Molina B Gonzalez de Pablo J Castillo A Diaz MA. Ruxolitinib Treatment for Steroid Refractory Acute and Chronic Graft *vs* Host Disease in Children: Clinical and Immunological Results. Am J Hematol (2019) 94:319–26. 10.1002/ajh.25376 30536806

[76] Modi B Hernandez-Henderson M Yang D Klein J Dadwal S Kopp E. Ruxolitinib as Salvage Therapy for Chronic Graft-*Versus*-Host Disease. Biol Blood Marrow Transplant (2019) 25:265–9. 10.1016/j.bbmt.2018.09.003 30201397

[77] Escamilla Gomez V Garcia-Gutierrez V Lopez Corral L Garcia Cadenas I Perez Martinez A Marquez Malaver FJ. Ruxolitinib in Refractory Acute and Chronic Graft-*Versus*-Host Disease: A Multicenter Survey Study. Bone Marrow Transplant (2020) 55:641–8. 10.1038/s41409-019-0731-x PMC7051903 31700138

[78] Uygun V Karasu G Daloglu H Ozturkmen S Kilic SC Yalcin K. Ruxolitinib Salvage Therapy is Effective for Steroid-Refractory Graft-*Versus*-Host Disease in Children: A Single-Center Experience. Pediatr Blood Cancer (2020) 67:e28190. 10.1002/pbc.28190 31981413

[79] Dang SH Liu Q Xie R Shen N Zhou S Shi W. Ruxolitinib Add-on in Corticosteroid-Refractory Graft-*vs*-Host Disease After Allogeneic Stem Cell Transplantation: Results From a Retrospective Study on 38 Chinese Patients. World J Clin Cases (2020) 8:1065–73. 10.12998/wjcc.v8.i6.1065 PMC7103980 32258077

[80] Yang W Zhu G Qin M Li Z Wang B Yang J. The Effectiveness of Ruxolitinib for Acute/Chronic Graft-*Versus*-Host Disease in Children: A Retrospective Study. Drug Des Devel Ther (2021) 15:743–52. 10.2147/DDDT.S287218 PMC7910527 33654380

[81] Mozo Y Bueno D Sisinni L Fernandez-Arroyo A Rosich B Martinez AP. Ruxolitinib for Steroid-Refractory Graft *Versus* Host Disease in Pediatric HSCT: High Response Rate and Manageable Toxicity. Pediatr Hematol Oncol (2021) 38:331–45. 10.1080/08880018.2020.1868637 33661711

[82] Wang YM Teusink-Cross A Elborai Y Krupski MC Nelson AS Grimley MS. Ruxolitinib for the Treatment of Chronic GVHD and Overlap Syndrome in Children and Young Adults. Transplantation (2021). 10.1097/TP.0000000000003768 33795598

[83] Hou C Dou L Jia M Li F Wang S Gao X. Ruxolitinib Combined With Corticosteroids as First-Line Therapy for Acute Graft-*Versus*-Host Disease in Haploidentical Peripheral Blood Stem Cell Transplantation Recipients. Transplant Cell Ther (2021) 27:75.e1–75.e10. 32961370 10.1016/j.bbmt.2020.09.015

[84] Zeiser R Socié G Schroeder MA Abhyankar S Vaz CP Kwon M. S256 Gravitas-301: A Randomized, Double-Blind Phase 3 Study of Itacitinib or Placebo in Combination With Corticosteroids for Initial Treatment of Patients With Acute Graft-Versus-Host Disease. HemaSphere (2020) 4:111–2. 10.1016/S2352-3026(21)00367-7 34971577

[85] Kroger N Shahnaz Syed Abd Kadir S Zabelina T Badbaran A Christopeit M Ayuk F. Peritransplantation Ruxolitinib Prevents Acute Graft-Versus-Host Disease in Patients With Myelofibrosis Undergoing Allogenic Stem Cell Transplantation. Biol Blood Marrow Transplant (2018) 24:2152–6. 10.1016/j.bbmt.2018.05.023 29800615

[86] Zhao Y Shi J Luo Y Gao F Tan Y Lai X. Calcineurin Inhibitors Replacement by Ruxolitinib as Graft-*Versus*-Host Disease Prophylaxis for Patients After Allogeneic Stem Cell Transplantation. Biol Blood Marrow Transplant (2020) 26:e128–33. 10.1016/j.bbmt.2020.01.012 31982545

[87] Morozova EV Barabanshikova MV Moiseev IS Shakirova AI Barhatov IM Ushal IE. A Prospective Pilot Study of Graft-*Versus*s-Host Disease Prophylaxis With Post-Transplantation Cyclophosphamide and Ruxolitinib in Patients With Myelofibrosis. Acta Haematol (2021) 144:158–65. 10.1159/000506758 32325461

[88] Choe H Shah NN Chevallier P Rubio MT Schroeder MA Hardy NM. Open-Label Phase 1 Study of Itacitinib (ITA) With Calcineurin Inhibitor (CNI)-Based Interventions for Prophylaxis of Graft-*Versus*-Host Disease (GVHD; GRAVITAS-119). Blood (2020) 136:50–1. 10.1182/blood-2020-140747

[89] Watkins B Qayed M McCracken C Bratrude B Betz K Suessmuth Y. Phase II Trial of Costimulation Blockade With Abatacept for Prevention of Acute GVHD. J Clin Oncol (2021) 39:1865–77. 10.1200/JCO.20.01086 PMC8260909 33449816

[90] Jagasia M Lazaryan A Bachier CR Salhotra A Weisdorf DJ Zoghi B. ROCK2 Inhibition With Belumosudil (KD025) for the Treatment of Chronic Graft-*Versus*-Host Disease. J Clin Oncol (2021) 39:1888–98. 10.1200/JCO.20.02754 PMC8189612 33877856

[91] Arora M Jagasia M Di Stasi A Meyers ML Quaranto C Schmitt A. Phase 1 Study of Axatilimab (SNDX-6352), a CSF-1r Humanized Antibody, for Chronic Graft-*Versus*-Host Disease After 2 or More Lines of Systemic Treatment. Blood (2020) 136:1–2. 10.1182/blood-2020-141553 32430499

